# Gene expression profiling of breast cancer in Lebanese women

**DOI:** 10.1038/srep36639

**Published:** 2016-11-18

**Authors:** Joelle Makoukji, Nadine J. Makhoul, Maya Khalil, Sally El-Sitt, Ehab Saad Aldin, Mark Jabbour, Fouad Boulos, Emanuela Gadaleta, Ajanthah Sangaralingam, Claude Chelala, Rose-Mary Boustany, Arafat Tfayli

**Affiliations:** 1 Department of Biochemistry and Molecular Genetics, American University of Beirut Medical Center, Beirut, Lebanon; 2 Department of Internal Medicine, University of Miami Miller SOM Regional Campus, 5301 S. Congress Avenue, Atlantis City, Fl 33462, US; 3 Department of Radiology, University of Iowa Hospitals and Clinics, 200 Hawkins Drive, Iowa City, IA 52242, US; 4 Department of Pathology and Laboratory Medicine, American University of Beirut Medical Center, Beirut, Lebanon; 5 Centre for Molecular Oncology, Barts Cancer Institute, Queen Mary University of London, Charterhouse Square, London EC1M 6BQ, UK; 6 Neurogenetics Program and Division of Pediatric Neurology, Department of Pediatrics and Adolescent Medicine, American University of Beirut Medical Center, Beirut, Lebanon; 7 Department of Internal Medicine, American University of Beirut Medical Center, Beirut, Lebanon

## Abstract

Breast cancer is commonest cancer in women worldwide. Elucidation of underlying biology and molecular pathways is necessary for improving therapeutic options and clinical outcomes. Molecular alterations in breast cancer are complex and involve cross-talk between multiple signaling pathways. The aim of this study is to extract a unique mRNA fingerprint of breast cancer in Lebanese women using microarray technologies. Gene-expression profiles of 94 fresh breast tissue samples (84 cancerous/10 non-tumor adjacent samples) were analyzed using GeneChip Human Genome U133 Plus 2.0 arrays. Quantitative real-time PCR was employed to validate candidate genes. Differentially expressed genes between breast cancer and non-tumor tissues were screened. Significant differences in gene expression were established for *COL11A1*/*COL10A1*/*MMP1/COL6A6/DLK1/S100P/CXCL11/SOX11/LEP/ADIPOQ/OXTR/FOSL1*/*ACSBG1* and *C21orf37*. Pathways/diseases representing these genes were retrieved and linked using PANTHER^®^/Pathway Studio^®^. Many of the deregulated genes are associated with extracellular matrix, inflammation, angiogenesis, metastasis, differentiation, cell proliferation and tumorigenesis. Characteristics of breast cancers in Lebanese were compared to those of women from Western populations to explain why breast cancer is more aggressive and presents a decade earlier in Lebanese victims. Delineating molecular mechanisms of breast cancer in Lebanese women led to key genes which could serve as potential biomarkers and/or novel drug targets for breast cancer.

Breast cancer (BC) is the most common cancer in women, accounting for 23% of all female cancers worldwide^1^. Globally, about 1.38 million women are diagnosed with BC and 458,503 die from the disease every year^2^. Also, BC incidence has shown an alarming increase worldwide^3^. In Lebanon, BC is the leading cancer among women^4^. Since early diagnosis and effective treatments are of great importance in reducing mortality of BC, researchers have been trying to identify additional biomarkers and novel drug targets^5^,^6^. An important risk factor is human epidermal growth factor (HER-2/neu)^7^. BC patients in Lebanon tend to present with an earlier age at diagnosis, 52 years compared to 63 years for counterparts in the West, including the United States^8^,^9^. This is consistent with data from Saudi Arabia^10^ and China^11^, providing additional evidence for the higher proportion of young BC in Asia than in Western countries. Usually, young BCs tend to have more aggressive biological behavior and clinical association with unfavorable prognosis compared with the disease arising in older women^12^. Young women with BC tend to have more advanced tumor TNM staging, more invasive pathological type, higher tumor grade, higher rates of lymph node positivity, higher proportion of triple-negativity, higher HER2 expression and lower ER/PR positivity^11^. Indeed, Lebanese BC patients tend to present with larger tumor size, more lymph node involvement, and higher tumor grades^9^. This may be accounted for by both environmental and genetic differences. A better understanding of the biology and molecular pathways for this disease is needed in order to tailor therapeutic options for this population.

Based on gene expression analysis, Perou *et al*. propose that BCs can be divided into the following classes: Luminal A [Estrogen Receptor Positive (ER+); low grade], luminal B (ER+; high grade), HER2 positive (*HER2*-amplification), and basal [ER−; Progesterone Receptor Negative (PR−); HER2−]^13^. These subclasses differ significantly in their prognosis and responsiveness to various therapeutic options.

Microarray technology is an effective tool to uncover global changes in the incidence and development of cancer^14^. The application of gene expression profiling is an excellent approach towards integrating the multiple molecular events and mechanisms by which cancer may develop^15^. The oncogenesis process involves the disruption of diverse cellular pathways including cell cycle, growth, survival and apoptosis. Gene expression microarrays allow the concurrent analysis of thousands of genes in a particular tumor providing a detailed description of the molecular fingerprint of the tumor.

In this study, differentially expressed genes (DEGs) from BC and normal tissue samples obtained at surgery were uncovered, allowing the extraction of a unique mRNA fingerprint of BCs in Lebanese women. PANTHER and Pathway Studio^®^ aided in constructing protein-protein interaction networks and highlighted significant pathways and novel target genes for the diagnosis of BC in Lebanese women. Moreover, characteristics of BCs in Lebanese women were compared to those of women from western populations in an attempt to shed light on why BC presents a decade earlier in Lebanese BC victims.

## Results

### Identification of differentially expressed genes (DEGs)

We used Affymetrix GeneChip Human Genome U133 Plus 2.0 expression arrays to determine differences in gene expression in 84 cases of invasive BC tissue compared to 10 non-tumor adjacent fresh breast tissue samples. Limma package in R was utilized to identify the DEGs between BC and non-tumor adjacent samples. Using an adjusted P < 0.05, 3053 genes were identified that were differentially expressed between tumor and non-tumor adjacent breast tissue. Among these, 1076 were up- and 1977 were down-regulated genes (see Supplementary Tables S1 and S2).

### Functional gene ontology analysis of breast tumor differentially expressed genes (DEGs)

**Figure 1:**
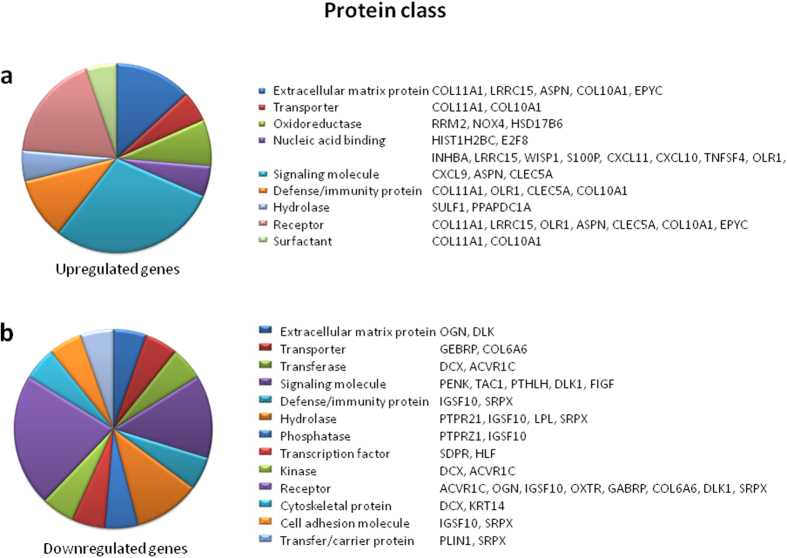
Pie chart representation of Gene Ontology (GO) for genes differentially expressed in microarray analyses and summarized according to protein class, in Lebanese population. Gene expression in tumor breast tissue was compared to surrounding non-tumor breast tissue, and the criterion for differential expression was choosing the top 50 fold changes with an adjusted P < 0.05, and a gene count >2 of the DEGs. Functional categories of DEGs were obtained using GO annotations from PANTHER classification system.

**Figure 2:**
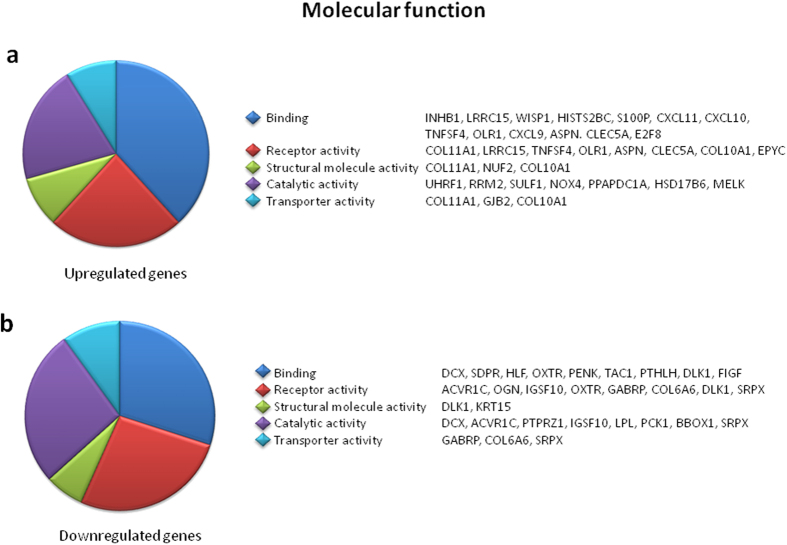
Pie chart representation of Gene Ontology (GO) for genes differentially expressed in microarray analyses and summarized according to molecular function, in Lebanese population. Gene expression in tumor breast tissue was compared to surrounding non-tumor breast tissue, and the criterion for differential expression was choosing the top 50 fold changes with an adjusted P < 0.05, and a gene count >2 of the DEGs. Functional categories of DEGs were obtained using GO annotations from PANTHER classification system.

To understand relevance of these genes, a program based on Gene Ontology (GO) categories of protein class and molecular function was employed. Figure 1 shows the main functional GO categories of transcripts that were differentially expressed between tumor and non-tumor breast tissue (adjusted P < 0.05; gene count >2 of the up- or down-regulated genes). Altered transcription of genes in the “protein class” category included a multitude of genes serving as signaling molecules or receptors (Fig. 1a,b; see Supplementary Table S3), and in the “molecular function” category, genes were mainly related to binding, receptor and catalytic activities (Fig. 2a,b; see Supplementary Table S3). The upregulated genes included Inhibin Beta A (*INHBA*), Wnt-1 Inducible Signaling Pathway Protein 1 (*WISP1*), S100 Calcium-Binding Protein P (*S100P*), Chemokine (C-X-C Motif) Ligand 9/10/11 (*CXCL9/10/11*), Collagen Type X Alpha 1 (*COL10A*1), Collagen Type XI Alpha 1 (*COL11A1*), Tumor Necrosis Factor Superfamily Member 4 (*TNFSF4*), NADPH Oxidase 4 (*NOX4*) and C-Type Lectin Domain Family 5 Member A (*CLEC5A*). The downregulated genes included Proenkephalin (*PENK*), Tachykinin Precursor 1 (*TAC1*), Delta-Like 1 Homolog (*DLK1*), c-Fos Induced Growth Factor (*FIGF*), Oxytocin Receptor (*OXTR*), Doublecortin (*DCX*), Parathyroid Hormone-Like Hormone (*PTHLH*), Protein Tyrosine Phosphatase Receptor-Type Z Polypeptide (*PTPRZ1*) and Collagen Type VI Alpha 6 (*COL6A*6).

### Protein-Protein Interaction (PPI) network of DEGs derived from breast cancer

Using a stringency ≥±2-log^2^ fold change in expression, the 3053 DEGs initially identified between tumor and non-tumor breast tissue were narrowed down, to only 170 DEGs, including 36 upregulated genes and 134 downregulated genes. To gain insight into how these DEGs affected cellular biological activity, a full overview of their interactions with each other was constructed to provide important clues of their functions. The gene or protein hubs with genes or proteins with high connectivity degree were identified (see Supplementary Fig. S1). A total of 8 gene hubs (red) were selected from the upregulated PPI network, which included *INHBA*, Matrix Metallopeotidase 12/13 (*MMP12/13*), *CXCL9/10/11, COL10A1* and Secreted Phosphoprotein 1 (SPP1). Meanwhile, 6 gene hubs (green), such as *DLK1, PTHLH, TAC1*, Adiponectin C1Q and Collagen Domain Containing (*ADIPOQ*), Leptin (*LEP*) and *FIGF*, were identified from the downregulated PPI network.

### Breast cancer DEGs common to all tumor grades

**Figure 3:**
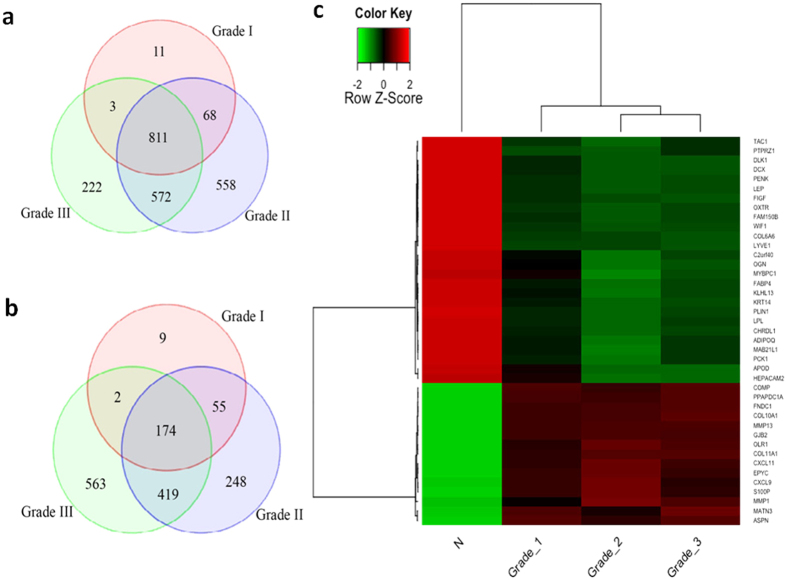
Differentially expressed genes shared by all tumor grades, in Lebanese population. Venn diagram displaying numbers of (**a**) upregulated and (**b**) downregulated DEGs in either grade I, grade II or grade III tumors. (**c**) 2-dimentional heat map of 41 DEGs. Heat map of mRNA abundance intensities of the differentially expressed genes in the profiled samples. RMA preprocessed data was transformed to z-scores. The legend represents relative over- (red) and under-expression (green). The labeling at the bottom represents the normal samples (N) and patients grouping by grade (1, 2 and 3). Adjusted P < 0.05, stringency ≥±3-log^2^ fold change in expression.

The data generated established significant differences in the expression of several genes in normal non-tumor adjacent samples vs. different tumor grades (grade I, II and III). Figure 3 demonstrates Venn diagrams (a and b) and a 2-D heat-map (c) of 41 DEGs considered significant only if they were up- or down-regulated by ≥±3-log^2^ fold change in response to cancer (adjusted P < 0.05). 2245 DEGs were upregulated in all 3 grades combined together (grade I, II and III), compared to only 1470 downregulated DEGs. Among these, 811 upregulated DEGs were common to all 3 grades, and only 174 DEGS were downregulated. Interestingly, grade II induced the highest number of upregulated DEGs, followed by grade III, and the lowest number of upregulated DEGs was induced by grade I (Fig. 3a). A number of Integrin signaling pathway DEGs including *COL11A1* and *COL10A1* were upregulated. *MMP1/13* were also robustly upregulated in all tumor grades, suggesting that BC may have led to an upregulation of genes involved in the plasminogen activating cascade and Alzheimer disease-presenilin pathway (Fig. 3c, adjusted P < 0.05). The largest number of downregulated DEGs was induced in grade III, followed by grade II and grade I tumors (Fig. 3b). In all tumor grades, a significant reduction in the expression of *COL6A6, DLK1, PENK* and *OXTR* involved in inflammation, angiogenesis, enkephalin release and oxytocin receptor mediated signaling pathway, respectively, were identified (Fig. 3c, adjusted P < 0.05).

### Expression of breast cancer-induced DEGs common to all molecular subtypes

**Figure 4:**
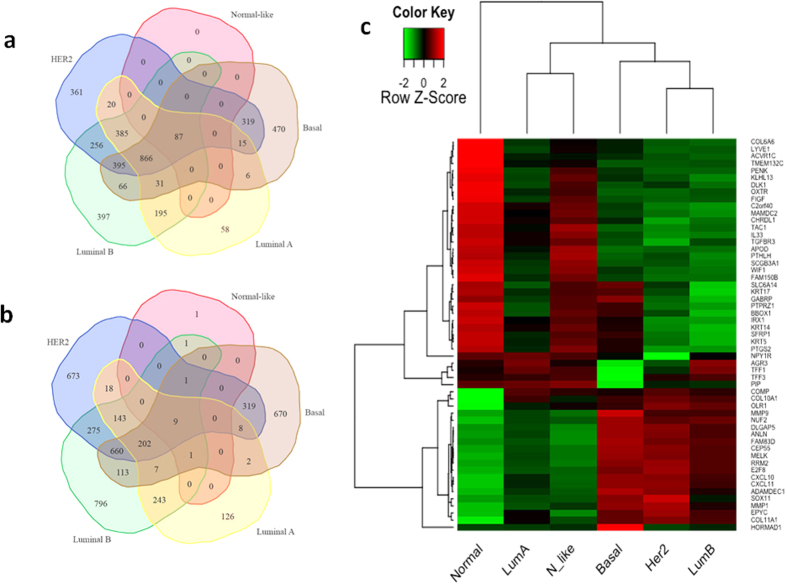
Differentially expressed genes shared by all molecular subtypes, in Lebanese population. Venn diagram displaying numbers of (**a**) upregulated and (**b**) downregulated DEGs in 5 different molecular subtypes (Normal-like, Basal, Luminal A, Luminal B and HER2). (**c**) 2-dimentional heat map of 55 DEGs. Heat map of mRNA abundance intensities of the differentially expressed genes in the profiled samples. RMA preprocessed data was transformed to z-scores. The legend represents relative over- (red) and under-expression (green). The labeling at the bottom represents the normal samples and patients grouping by PAM50 molecular subtype. Adjusted P < 0.05, stringency ≥±3-log^2^ fold change in expression.

Similarly, the data established significant differences in the expression of several genes in normal non-tumor samples vs. different molecular subtypes (Normal-like, Basal, Luminal A, Luminal B and HER-2). Figure 4 shows Venn diagrams (a and b) and 2-D heat-maps (c) of 55 DEGs considered significant if they were up- or down-regulated by ≥±3-log^2^ fold change in response to cancer (adjusted P < 0.05). 3927 DEGs were upregulated in all 5 molecular subtypes combined together (Normal-like, Basal, Luminal A, Luminal B and HER-2), compared to 4262 downregulated DEGs. Among these, a total of 87 upregulated DEGs were common to all 5 molecular subtypes, and only 9 DEGS were downregulated in the 5 molecular subtypes. Interestingly, HER-2 and Luminal B types induced the highest number of up- or down-regulated DEGs, followed by Basal and Luminal A. Not surprisingly, the lowest number of upregulated DEGs was induced in Normal-like subtype (Fig. 4a). Integrin signaling pathway DEGs included *COL11A1* and *COL10A1. MMP1/9* were also robustly upregulated in 4 molecular subtypes (Luminal A, Luminal B, HER2 and Basal), suggesting that BC may have led to an upregulation of genes involved in the Alzheimer disease-presenilin pathway (Fig. 4c, adjusted P < 0.05). The highest number of downregulated DEGs was induced in HER-2 and Luminal B types, followed by Basal and Luminal A. The lowest number of downregulated DEGs was observed in Normal-like subtype (Fig. 4b). A significant reduction in the expression of *COL6A6* and Prostaglandin-Endoperoxide Synthase 2 (*PTGS2*) involved in inflammation and in *DLK1* and Secreted Frizzled-Related Protein 1 (*SFRP1*) both involved in angiogenesis was observed in the 4 molecular subtypes,. As expected, the expression of significant DEGs was very similar between the Normal-like subtype and non-tumor adjacent samples (Fig. 4c, adjusted P < 0.05).

### Validation of microarray analysis results with qRT-PCR

**Figure 5:**
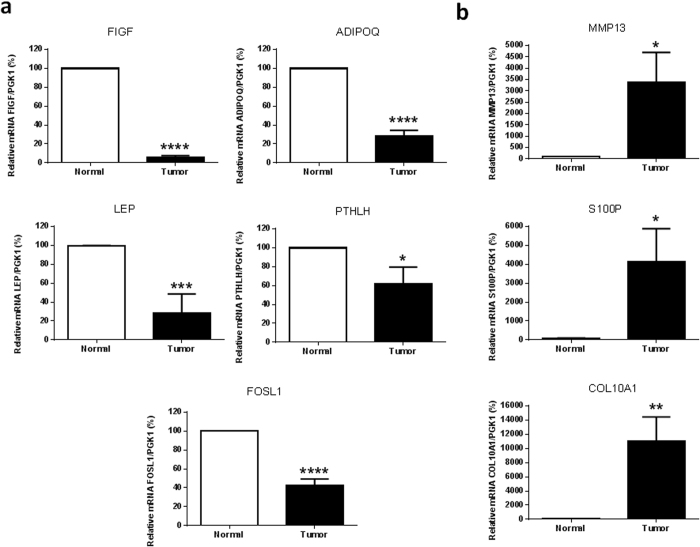
Validation of microarray analysis results with qRT-PCR, in Lebanese population. Relative mRNA expression was measured by quantitative RT-PCR. (**a**) The following underexpressed genes were randomly chosen: *FIGF* (P < 0.0001), *ADIPOQ* (P < 0.0001), *LEP* (P = 0.0009), *PTHLH* (P = 0.0345) and *FOSL1* (P < 0.0001). (**b**) The following overexpressed genes were randomly chosen: *MMP13* (P = 0.0169), *S100P* (P = 0.0234) and *COL10A1* (P = 0.024). Values are means of the fold changes normalized to *PGK1* mRNA expression, with their standard errors represented by vertical bars. *P < 0.05, **P < 0.01, ***P < 0.001, ****P < 0.0001 by Student’s t-test (n = 27).

Seven genes were randomly chosen for further analysis by qRT-PCR based on biological relevance. Results exhibited consistency with those of microarray analysis, validating our findings. Tumor status induced a significant decrease in the expression of *FIGF* (P < 0.0001), *ADIPOQ* (P < 0.0001), *LEP* (P = 0.0009), *PTHLH* (P = 0.0345) and FOS-like Antigen 1 (*FOSL1*; P < 0.0001) compared with expression in corresponding non-tumor tissue (Fig. 5a). There was also a significant increase in the expression of *MMP13* (P = 0.0169), *S100P* (P = 0.0234) and *COL10A1* (P = 0.024) compared with expression in the corresponding non-tumor tissue (Fig. 5b).

### Comparison analysis of DEGs in Lebanese and Western populations

**Figure 6:**
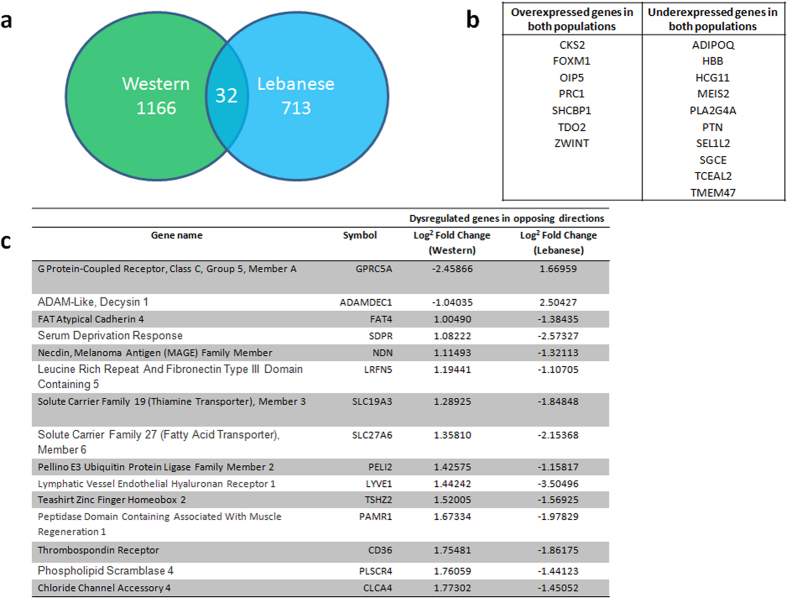
Comparative analysis of DEGs in Western and Lebanese datasets. In order to compare the difference of DEGs selected in Western and Lebanese populations datasets, gene expression in tumor breast tissue was compared to surrounding non-tumor breast tissue in both populations, and the criterion for differential expression was an adjusted P < 0.05, and a stringency ≥±1-log^2^ fold change in expression. (**a**) A total number of 1911 genes were left, among which 1166 and 713 genes were exclusive for the Western and Lebanese populations, respectively. Only 32 genes were found in the overlapping region, so were common to both datasets. (**b**) List of the 17 genes that are commonly over- or underexpressed in both populations. (**c**) List of the 15 genes and their log^2^ fold changes that are differentially expressed in both populations, but in opposing directions.

Limma package in R was utilized to identify the DEGs between BC and non-tumor adjacent samples, in Lebanese and Western populations. Using an adjusted P < 0.05 and a stringency ≥±1-log^2^ fold change in expression, 1911 significant genes were identified that were differentially expressed between tumor and non-tumor breast tissue in both population datasets. Thirty-two genes found in the overlapping region were common to the two datasets, implying that these genes were differentially expressed in both Lebanese and Western patients (Fig. 6a). Seventeen of these 32 genes were commonly over- or underexpressed in both populations (Fig. 6b), whereas the remaining 15 genes were dysregulated in opposing directions. Among those 15 genes, the most commonly highly up- or downregulated were G Protein-Coupled Receptor, Class C, Group 5, Member A (*GPRC5A*), Peptidase Domain Containing Associated With Muscle Regeneration 1 (*PAMR1*), Teashirt Zinc Finger Homeobox 2 (*TSHZ2*), Lymphatic Vessel Endothelial Hyaluronan Receptor 1 (*LYVE1*) and Thrombospondin Receptor (*CD36*) (Fig. 6c). There were 1879 differentially expressed candidate genes appearing exclusively in either Western or Lebanese patients, as depicted by the Venn diagram (Fig. 6a). Among these, 1166 and 713 DEGs were exclusive genes in either Western or Lebanese populations, respectively. These mutually exclusive genes drew our attention since they might reflect the differences between the two populations, and may potentially influence the BC in the two populations. Further details about the most significant exclusive genes in each population are shown in Supplementary Tables S1, S2 and S4.

## Discussion

Early detection and accurate diagnosis are an effective method to lower mortality of BC. The identification of molecular subtypes and the development of prognostic and predictive molecular signatures through gene expression profiling have resulted in a better appreciation of the biologic heterogeneity of BC^16^.

mRNA expression levels in BC fresh tissue samples were determined and association with patient clinical characteristics examined. 3053 DEGs including 1076 up-regulated and 1977 down-regulated DEGs were identified. Classification of Lebanese tumor samples was assessed by differences in gene expression patterns into different molecular subtypes^13^: Luminal A (35%), luminal B (24%), HER2 positive (17%), basal (14%) and normal-like (9%). A distribution of breast cancer subtypes is similar to what is reported in western populations^17^. Lebanese women have tumors with similar gene expression patterns in luminal A, luminal B, basal and HER2 subtypes to those in women in the west. GATA-binding protein 3 (*GATA3*) and Estrogen Receptor 1 (*ESR1*) were significantly upregulated in Lebanese luminal A and B, the only ER+ subtypes. Keratins 8/18 (*KRT8/18*), characteristic of luminal epithelial cells, are significantly overexpressed in Lebanese luminal B tumors. Keratins 5/17 (*KRT5/17*) characteristic of basal epithelial cells are significantly under-expressed in Lebanese luminal A/B tumors. *HER2/neu* or *Erb-B2* is significantly overexpressed in Lebanese HER2 positive breast tumors. Keratin 6 (*KRT6*) was significantly up-regulated in Lebanese basal subtype, whereas *KRT8, GATA3* and X-box binding protein 1 (*XBP1*) were significantly down-regulated.

**Figure 7:**
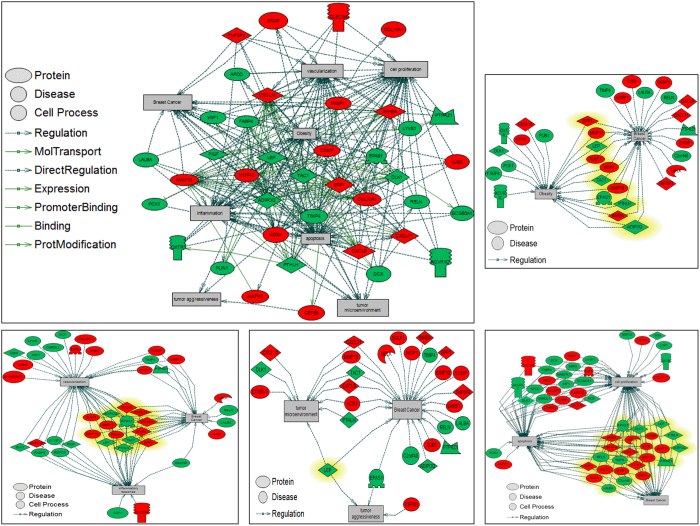
Functional relationship network of DEGs between tumor and non-tumor adjacent breast tissue, in Lebanese population. Pathway Studio^®^ generated network interactions between most significant DEGs in breast tissue (Adjusted P < 0.05, stringency ≥±2-log^2^ fold change in expression). Upregulated genes are designated in red and downregulated genes in green.

Most up-regulated DEGs were involved in pathways of Integrin signaling and presenilin. Down-regulated DEGs were involved in inflammation and angiogenesis. *COL11A1, COL10A1, MMP1, COL6A6* and *DLK1* genes were identified in the Lebanese cohort (Fig. 7).

A number of genes differentially expressed (non-tumor adjacent tissue vs. BC tissue) are related to extracellular matrix (ECM), including *COL11A1, COL10A1* and *COL6A6*. These proteins maintain integrity of tissues including muscle, tendons, skin, cartilage, and intervertebral disks^18^. In invasive carcinomas, extracellular collagens are key to tumor behavior and are subject to continuous remodeling, inhibiting and/or promoting tumor progression, according to stage of tumor development^19^. Related pathways are PI3K-Akt and ERK signaling. *COL11A1* encodes a minor collagen found in many tissues and dysregulated in breast and colon cancer^20^. *COL10A1* encodes a short chain collagen expressed by hypertrophic chondrocytes during endochondral ossification. Its expression is elevated in diverse solid tumor types and is associated with tumor vasculature^21^. Tissues analyzed demonstrated significant up-regulation of *COL11A1* and *COL10A1* in tumor tissue as compared to non-tumor adjacent tissue in the Lebanese, suggesting that both proteins might play a role in local invasion of BC cells. Altered expression of *COL11A1/COL10A1* is associated with tumor development/progression. Several studies reported that *COL11A1* is overexpressed in invasive ductal carcinoma (IDC) of the breast relative to ductal carcinoma *in situ* (DCIS) in Spanish populations. This overexpression is correlated with carcinoma aggressiveness, progression, and lymph node metastasis^22^. Chang *et al*. report that *COL10A1, MMP13, CAMP* and *FLJ25416* are overexpressed in human BC tissues in the Taiwanese^23^. Collagen VI (COL6) up-regulation is reported in various aspects of tumor progression. Several malignant cancer cell lines express COL6 including those derived from mammary gland, colon, pancreatic ductal adenocarcinomas and hepatocarcinoma^24^. *COL6A6*, a form of COL6, is expressed in a wide range of fetal and adult brain, heart and muscle^25^. *COL6A6* is increased in Ullrich congenital muscular dystrophy^26^. *COL6A6* is downregulated in tumor tissue versus adjacent normal breast tissue in the Lebanese.

Human MMP proteins include 24 members^27^ function in breakdown of extracellular matrix in normal physiological processes of embryonic development, wound healing, and tissue remodeling, and play an important role in arthritis, cancer infiltration, metastasis and angiogenesis^23^. *MMP1* (collagenase-1)/*MMP8* (collagenase-2)/*MMP13* (collagenase-3) can break down collagen types I, II, III and V. *MMP13* is overexpressed in human BC tissues, in Danish and Taiwanese populations, and imply that in the process of BC turning from ductal carcinoma *in situ* to invasive ductal carcinoma, *MMP13* can break down basement membranes resulting in invasive cancer^23^,^28^. *MMP1* is highly expressed in many types of cancer, specifically BC in Western populations, and positively correlates with accelerated cell migration, advanced clinical stage and metastasis in BC, making *MMP1* a significant predictor of poor prognosis in BC^29^.

*DLK1*, a member of the EGF-like repeat-containing family of proteins, includes NOTCH receptors and ligands. DLK1 is involved in differentiation, cell proliferation and tumorigenesis. DLK1 may function both as oncogenic and anti-oncogenic^30^. Kawakami *et al*. indicate that expression of DLK1 was lost in primary renal cell carcinoma (RCC) compared to adjacent normal kidney tissue in the Japanese and show tumor growth to be blocked by restoration of DLK1 compared to control cells suggesting that DLK1 has tumor suppressor activity in RCCs *in vitro* and *in vivo*^31^. Human DLK1 was also expressed in colon, breast, pancreas and lung carcinoma *in vitro*^32^.

Our data also demonstrates significant up-regulation of *COL11A1, COL10A1, MMP1* and *MMP13*, and significant down-regulation of *COL6A6* and *DLK1* in tumor relative to non-tumor adjacent breast tissue. *COL11A1, COL10A1, MMP1* and *MMP13* are highly expressed in aggressive molecular subtypes (basal and HER2 tumors) in the Lebanese as their expression increases tumor migration and proliferation. *COL11A1, COL10A1, MMP1*, and *DLK1* are known to play a role in BC and tumorigenesis, but not *COL6A6*. So, COL6A6 may be a potential novel biomarker for BC in Lebanese women. Further studies are needed before considering expression of these genes in decisions of treatment and whether considering them as targets for therapy or as prognostic factors.

Significant differences in tumor vs. non-tumor adjacent tissue regarding expression of several other genes were established in the Lebanese. *S100P, CXCL11*, and SRY (Sex Determining Region Y)-Box 11 (*SOX11*) display higher expression in tumor samples. *LEP, ADIPOQ*, and *OXTR* manifest lower expression in tumor samples.

Several studies from Germany reported overexpression of *S100P* in BC, among other cancers^33^. In addition to their function as mediators of inflammatory responses, chemokines are involved in the regulation of tumor development and metastasis. Levels of the three chemokines *CXCL9, CXCL10* and *CXCL11* were markedly higher in metastatic BC patients as compared to healthy control sera, in the West^34^. Liu *et al*. demonstrated that expression of *SOX11* in BC tissues was significantly higher than in normal tissue, in the Chinese. This overexpression is correlated with smaller tumor size and milder tumor grade, indicating that SOX11 could inhibit growth and progression of BC, and promote absence of lymph node metastasis^35^. Both *LEP* and *ADIPOQ* are adipokines secreted by adipocytes. *LEP* has been directly associated with obesity, while *ADIPOQ* has been inversely associated with obesity and visceral fat accumulation. Basu *et al*. demonstrated that both *LEP* and *ADIPOQ* are expressed at higher levels in normal adjacent breast tissue compared to tumor specimens, in the French population^36^. The hormone oxytocin is common in cells of healthy breast tissue, but is rarely or never detected in BC *in vitro* and *in vivo*. Oxytocin inhibits proliferation of human BC cell lines, and thus may play a role in preventing this disease^37^.

In clinical practice, the elucidation of the association between gene expression profiles and clinico-pathological characteristics may aid physicians in selecting patient-suitable treatments. Good biomarkers can aid in early cancer screening, confirm cancer diagnosis, predict outcome, tailor therapy, and guide future research directions by shedding light on new tumorigenesis pathways. In this study, when comparing gene expression between BC tissue and adjacent non-tumor tissue, no significant association with the following clinical variables was determined: age, menopausal status, lower or higher tumor grade, molecular subtype, HER2 expression, or family history of BC (see Supplementary Figs S2 and S3). Whereas, our findings confirm a significant association in Lebanese BC patients between the expression levels of *FOSL1*, Acyl-CoA synthetase bubblegum family member 1 (*ACSBG1*), and Chromosome 21 open reading frame 37 (*C21orf37*) genes with ER/PR expression and family history of ovarian cancer. This suggests the possible usefulness of including them as biomarkers in BC diagnostics. The 3 genes (*FOSL1, ACSBG1*, and *C21orf37*) were downregulated in our patients with ER-negative BCs (log^2^-fold change: −0.37; −0.16; −0.34; adjusted P < 0.05), and PR-negative BCs (log^2^-fold change: −0.28; −0.15; −0.18; adjusted P < 0.05); and upregulated in patients having a family history of ovarian cancer (log^2^-fold change: 1.08; 0.5; 0.8; adjusted P < 0.05). Hormone receptor-negative tumors have a higher chance of recurrence than hormone receptor-positive tumors in the first five years after diagnosis. After five years, this difference decreases and over time, goes away^38^. Having a family history of ovarian cancer increases the chances of getting breast or ovarian cancer. Such knowledge has great potential in preventive strategies, particularly for women with family history of ovarian cancer who are at high risk, and for women with ER/PR-negative BCs. *FOSL1* is downregulated in breast tumor tissues, in our cohort. In high-grade cases of BC tissue, low expressions of genes involved in apoptosis, cell cycle control and immune pathways are reported. These include the *FOSL1* gene^39^. Acyl-CoA synthetases (ACSs) that catalyze the conversion of fatty acids to their active form acyl-CoAs play a central role in fatty acid metabolism. ACS bubblegum (*ACSBG*) is one subfamily containing *ACSBG1* and *ACSBG2* isozymes^40^. Several studies suggest that *ACSBG1* may be involved in the biochemical pathology of X-linked adrenoleukodystrophy (X-ALD). Patients with X-ALD have neurodegeneration and elevated serum and tissue levels of saturated very long-chain fatty acids. In mice, *ACSBG1* is highly expressed in the brain, adrenal cortex, and testis^41^. We suggested, for the first time, the involvement of the *ACSBG1* gene in BC in Lebanese cases, making it a potential novel gene for BC prognosis in Lebanese women. Also, nothing is known about *C21orf37* gene and its association with cancer, making it a possible gene or biomarker in BC in the Lebanese. Further investigations are needed to validate whether these genes can serve as targets for therapy or as prognostic factors in Lebanese BC.

Our comparative analysis showed that most of the genes exclusively dysregulated in Western populations were involved in signaling pathways of Integrin and inflammation mediated by chemokine and cytokine. Genes including LIM and Senescent Cell Antigen-Like Domains 1 (*LIMS1*) and Platelet Factor 4 Variant 1 (*PF4V1*) were significantly identified in these pathways (see Supplementary Table S4). *LIMS1*, a protein containing 5 LIM domains or double zinc fingers, plays a role in integrin-mediated cell adhesion or spreading. It is also known to be involved in the regulation of cell survival, cell proliferation and cell differentiation. *LIMS1* was significantly upregulated in tumor tissue in Western populations, which is in agreement with its anti-apoptotic role in cancer (log^2^-fold change: 3.05; adjusted P < 0.05). Angiogenesis, the formation of an ever more branching network of blood vessels from a pre-existing vascular network, has been established as a requisite for successful tumor growth and cancer progression^42^. *PF4V1* also known as *CXCL4L1*, displays a strong anti-angiogenic function and thus impairs tumor growth. In fact, Van Raemdonck *et al*. showed that platelet-derived CXC chemokines, *CXCL4* and *CXCL4L1*, inhibited lymphatic endothelial cell proliferation *in vitro*, reduced proliferation of MDA-MB-231 cells *in vitro* and decreased MDA-MB-231 tumor growth *in vivo*^43^. The anti-tumoral activity of *PF4V1* via inhibition of angiogenesis and induction of apoptosis within tumor tissue was confirmed by the significant downregulation of this gene expression in tumor tissue of Western populations (log^2^-fold change: −3.20; adjusted P < 0.05).

Nevertheless, among the exclusively dysregulated genes in the Lebanese population, we mention *COL11A1, COL10A1, MMP1* and *MMP13* that were significantly upregulated in breast tumor tissue compared to adjacent non-tumor tissue, making BC in this population more aggressive with increased tumor migration and proliferation. Also, levels of the three chemokines *CXCL9, CXCL10* and *CXCL11* were markedly higher, exclusively in Lebanese tumor tissue as compared to non-tumor breast tissue, making BC in this population prone to metastasis (see Supplementary Tables S1 and S2).

Of the 17 genes that were significantly overexpressed in both Western and Lebanese populations, we mention Cyclin Kinase Subunit 2 (*CKS2*), Forkhead Box M1 (*FOXM1*), and Src Homolog and Collagen Homolog Binding Protein 1 (*SHCBP1*) (Fig. 6b). CKS2 protein is essential for the first metaphase/anaphase transition of mammalian meiosis. It is known to be up-regulated in various malignancies including breast cancer tissue, suggesting that *CKS2* may be an oncogene^44^. FOXM1 is ubiquitously expressed in cells undergoing proliferation, and overexpression of *FOXM1* is associated with poor prognosis in various malignant tumors including breast cancers^45^. *SHCBP1* is shown to be significantly up-regulated in breast cancer tissues compared with adjacent normal tissues, and its overexpression is correlated with advanced clinical stage and poorer survival^46^. Whereas, Adiponectin (*ADIPOQ*) and Sarcoglycan Epsilon (*SGCE*) were significantly under-expressed in both populations (Fig. 6b). Basu *et al*. demonstrated that *ADIPOQ* is expressed at higher levels in normal adjacent breast tissue compared to tumor specimens^36^. Ortega *et al*. demonstrated that *SGCE* is significantly downregulated in colorectal tumors^47^.

Of the 15 genes with opposite dysregulation in both populations, we mention Teashirt Zinc Finger Homeobox 2 (*TSHZ2*) and Peptidase Domain Containing Associated with Muscle Regeneration 1 (*PAMR1*) that were significantly upregulated in Western populations but significantly downregulated in the Lebanese (Fig. 6c). Carcinogenesis is the result either of the activation of proto-oncogenes or the inactivation of tumor suppressor genes. Yamamoto *et al*. showed, for the first time, that Teashirt Zinc Finger Homeobox 2 (*TSHZ2*) exhibited consistent diminished expression in many cancer cell lines compared to normal epithelial cells and in 100% of the BC clinical cases examined, making it a novel tumor suppressor gene^48^. Similarly, a recent study showed that Peptidase Domain Containing Associated with Muscle Regeneration 1 (*PAMR1*) expression was reduced in all tested BC cell lines, while it was expressed moderately in normal breast tissues and primary mammary epithelial cells. In addition, ectopic expression of *PAMR1* markedly suppressed cancer cell growth, making it a putative BC tumor suppressor^49^. The downregulation of these two genes may also be observed in several other types of cancers, including prostate cancer. So, the suppression of certain tumor suppressor genes such as *TSHZ2* and *PAMR1* may be the cause of the more aggressive type of BC in Lebanese patients, leading to poor prognosis and an increased risk of death.

On the contrary, G Protein-Coupled Receptor, Class C, Group 5, Member A (*GPRC5A*) was found to be upregulated in Lebanese but under-expressed in Western populations (Fig. 6c). *GPRC5A* is overexpressed in breast, colon and gastric cancers^50^, with the caveat that *GPRC5A*, among 80 genes, was significantly up-regulated in young BC patients (aged ≤45 years) relative to older, post-menopausal BCs (aged ≥65 years) (Fold change: 0.67; adjusted P < 0.01)^11^. When comparing age of diagnosis, the Lebanese population had a larger number of young patients, which may explain the upregulation of *GPRC5A* in our population. More interestingly, a set of genes such as *CXCL* and *MMP*, were also up-regulated in young patients with BC, in Asian population^11^. Their important roles in cancer cell migration, invasion, and metastasis have been documented^51^. As mentioned, *MMP1, MMP13, CXCL9, CXCL10* and *CXCL11* were significantly upregulated, exclusively in Lebanese tumor tissue compared to adjacent non-tumor tissue (see Supplementary Tables S1 and S2), explaining why Lebanese patients diagnosed at a younger age are prone to develop more aggressive BCs with a greater potential for metastasis associated with poor prognosis.

Our findings may support the notion that Lebanese patients are exposed to unique environmental/genetic factors. Further investigations are necessary to explore influences other than genetic factors that may correlate with behavior of BC in different countries.

In conclusion, several genes associated with BC have been used as diagnostic or prognostic markers, and/or therapeutic targets worldwide, such as HER2^52^,^53^. Biomarkers for early diagnosis and therapy of BC are still limited. Gene expression microarrays have allowed identification of a differentially regulated subset of genes which may play a role in the development of BC in Lebanese women. Findings suggest that expression of functionally related genes and transcription factors are modulated in BC in a population-specific manner, with wider implications for all BC patients. Informed speculations about other mechanisms underlying tumorigenesis have emerged. Further elucidation of signaling pathways linking genes to tumor behavior may provide key insights into molecular mechanisms driving breast tumorigenesis.

## Materials and Methods

### Subjects and biological sampling

The research protocol was approved by the Institutional Review Board (IRB) at the American University of Beirut (AUB), all subjects provided written informed consent, and all methods were conducted in accordance with approved guidelines and regulations. Subjects were recruited from American University of Beirut Medical Center (AUBMC), between September 2012 and May 2014. The sample consisted of 84 subjects (aged 33–84 years) and included female individuals newly diagnosed with stage I, II or III BC that were subjected to surgical resection at AUBMC. Exclusion criteria included prior history of malignancies or any therapy for BC. Fresh BC and corresponding non-tumor breast samples were also collected and stored in RNA*later*^TM^ (Qiagen) at −20 °C, until further analysis. Normal tissue controls were selected to be as far away as possible from the malignant tumor from mastectomy specimens.

### Clinical variables

Clinical covariates used in gene expression analysis include age, menopausal status, tumor grade, stage, molecular subtype, estrogen receptor (ER) status, progesterone receptor (PR) status, human epidermal growth factor receptor 2 (HER2) status, and family history of other cancers (including breast and ovarian cancer). Clinical characteristics of the patients are summarized in Supplementary Table S5.

### RNA isolation

Using RNeasy^®^ Plus Mini Kit (Qiagen), total RNA was isolated from human fresh breast tissues, according to the manufacturer’s protocol and stored at −80 °C. For assessing RNA quality and yield, A_260_/A_280_ and A_260_/A_230_ ratios for RNA were analyzed with Experion^TM^ Automated Electrophoresis System (BioRad). RNA concentrations were determined by absorption at 260 nm wavelength with a ND-1000 spectrometer (Nanodrop Technologies).

### Gene expression analysis

A total of 94 fresh breast tissue samples, comprising 84 cancer samples and 10 adjacent non-tumor samples, were collected and used to represent the Lebanese cohort. Gene expression profiling was performed using the GeneChip Human Genome U133 Plus 2.0 arrays (Affymetrix Inc.) representing over 45,000 transcripts. Briefly, 100 ng of total RNA were amplified, labeled, fragmented and then hybridized using the GeneChip 3′ IVT Express Kit as instructed by the manufacturer. After washing and staining, using the GeneChip Fluidics Station 450, the arrays were scanned with a GeneChip Scanner 3000 7G. Cell Intensity Data (CEL) files were generated using the Affymetrix GeneChip Command Console (AGCC) software version 3.2. Data used to represent the Western cohort were downloaded from the NCBI GEO database^54^. These comprised raw data files from two Affymetrix datasets - GSE65194^55^ and GSE2568^56^.

All data analyses were performed in R (v3.2.2), using Bioconductor (http://www.bioconductor.org) and associated packages. After the application of stringent quality control criteria, the data from each cohort was quantile normalized independently, using the Robust Multi-array Average (RMA) algorithm^57^. Differential expression analysis between the tumor and normal expression profiles were conducted. First, the mRNA abundance profiles of probes representing individual genes were collapsed for each patient, with the mean value used to represent the gene expression summary. Genes reported differentially expressed (DE) between the tumor and normal samples from each cohort were identified using limma^58^, which fits a gene-wise linear model to the normalized expression values. In addition, the Benjamini and Hochberg’s method was applied to control the false discovery rate (FDR)^59^, with significance thresholds being set at FDR ≤0.05 and an absolute log^2^ fold change of at least 1. Subsequently, the lists of DE gene generated from the Lebanese and Western analyses were compared.

Heat maps and Venn diagrams were generated using the R packages gplot and VennDiagram, respectively. PANTHER^®^ version 10.0 and Pathway Studio^®^ were used for detection of gene ontology and canonical pathway analysis with significant transcripts in tumor group compared to control. Networks of biologically related genes were created using Pathway Studio^®^.

### Quantitative real-time PCR

Confirmation of microarray results was performed by quantitative real-time PCR. Total RNA was reverse transcribed using RevertAid Reverse Transcriptase (Thermo Scientific) with 100–1000 ng of input RNA and random primers (Thermo Scientific). Quantitative real-time PCR reactions were performed in 96-well plates using specific primers (TIB MOLBIOL) and the iQ^TM^ SYBR^®^ Green Supermix (BioRad) as a fluorescent detection dye, in CFX96^TM^ Real-Time PCR (BioRad), in a final volume of 12.5 μl. To characterize generated amplicons and to control contamination by unspecific by-products, melt curve analysis was applied. Each reaction was performed in duplicate. All results were normalized to *PGK1* mRNA level and calculated using the ΔΔC_t_ method. The specificity of the PCR was determined by melt-curve analysis for each reaction. Primer sequences are listed in Supplementary Table S6.

### Statistical analysis

mRNA expression was quantified in breast adenocarcinomas compared to normal tissue. Continuous data was expressed as means ± SEM, and compared by the two-tailed Student’s *t-*test. Gene expression data derived by Affymetrix or quantitative real-time PCR was compared for tumor vs. normal non-tumor adjacent tissue by standard *t*-test.

GraphPad Prism 6 (GraphPad Software, Inc., California, USA) was used for analysis, except for the Affymetrix expression data that was analyzed using R statistical environment. All tests were two-sided and an adjusted P < 0.05 was considered as a statistically significant difference.

## Additional Information

**How to cite this article**: Makoukji, J. *et al*. Gene expression profiling of breast cancer in Lebanese women. *Sci. Rep.* **6**, 36639; doi: 10.1038/srep36639 (2016).

**Publisher’s note:** Springer Nature remains neutral with regard to jurisdictional claims in published maps and institutional affiliations.

## Supplementary Material

Supplementary Information
