# Supplementary material for: Gene expression profiling of breast cancer in Lebanese women
Source: Sci Rep. 2016 Nov 18;6:36639. doi: 10.1038/srep36639 (PMC5114572; doi:10.1038/srep36639)
Supplement: Supplementary Information [file srep36639-s1.pdf]

Gene expression profiling of breast cancer in Lebanese women

Joelle Makoukji[1], Nadine J. Makhoul[1], Maya Khalil[2], Sally El-Sitt[1], Ehab Saad Aldin[3], Mark Jabbour[4], Fouad Boulos[4], Emanuela Gadaleta[5], Ajanthah Sangaralingam[4], Claude Chelala[5], Rose-Mary Boustany[¶*1,6], Arafat Tfayli[¶7]

Gene Name	Symbol	log^2 Fold Change
Collagen, Type X, Alpha 1	COL10A1	5.14
Collagen, Type XI, Alpha 1	COL11A1	4.62
Gap Junction Protein, Beta 2, 26kDa	GJB2	4.49
Phosphatidic Acid Phosphatase Type 2 Domain Containing 1A	PPAPDC1A	4.01
Matrix Metallopeptidase 1	MMP1	3.64
Fibronectin Type III Domain Containing 1	FNDC1	3.45
Cartilage Oligomeric Matrix Protein	COMP	3.43
S100 Calcium Binding Protein P	S100P	3.25
Epiphycan	EPYC	3.20
Chemokine (C-X-C Motif) Ligand 11	CXCL11	2.88
Oxidized Low Density Lipoprotein (Lectin-Like) Receptor 1	OLR1	2.87
Chemokine (C-X-C Motif) Ligand 9	CXCL9	2.75
Matrix Metallopeptidase 13	MMP13	2.67
Hydroxysteroid (17-Beta) Dehydrogenase 6	HSD17B6	2.62
Inhibin, Beta A	INHBA	2.52
Asporin	ASPN	2.51
Matrilin 3	MATN3	2.45
Chemokine (C-X-C Motif) Ligand 10	CXCL10	2.42
Histone Cluster 1, H2bc	HIST1H2BC	2.33
C-Type Lectin Domain Family 5, Member A	CLEC5A	2.31
Tumor Necrosis Factor (Ligand) Superfamily, Member 4	TNFSF4	2.31
Collagen Triple Helix Repeat Containing 1	CTHRC1	2.27
Sulfatase 1	SULF1	2.17
NUF2, NDC80 Kinetochore Complex Component	NUF2	2.16
Chromosome 3 Open Reading Frame 80	C3orf80	2.13
Ribonucleotide Reductase M2	RRM2	2.06
Centrosomal Protein 55kDa	CEP55	2.01
Anillin, Actin Binding Protein	ANLN	2.00
Secreted Phosphoprotein 1	SPP1	1.98
Leucine Rich Repeat Containing 15	LRRC15	1.97
E2F Transcription Factor 8	E2F8	1.96
Ubiquitin-Like With PHD And Ring Finger Domains 1	UHRF1	1.94
Maternal Embryonic Leucine Zipper Kinase	MELK	1.94
NADPH Oxidase 4	NOX4	1.89
Synapse Differentiation Inducing 1	SYNDIG1	1.81
WNT1 Inducible Signaling Pathway Protein 1	WISP1	1.73
G Protein-Coupled Receptor, Class C, Group 5, Member A	GPRC5A	1.67
Lysosomal-Associated Membrane Protein Family, Member 5	LAMP5	1.67
Cyclin E2	CCNE2	1.67
TPX2, Microtubule-Associated	TPX2	1.66
Ubiquitin-Conjugating Enzyme E2T	UBE2T	1.65
Radical S-Adenosyl Methionine Domain Containing 2	RSAD2	1.64
Kinesin Family Member 4A	KIF4A	1.64
Denticleless E3 Ubiquitin Protein Ligase Homolog	DTL	1.62
BCL2-Related Protein A1	BCL2A1	1.57
Triggering Receptor Expressed On Myeloid Cells 1	TREM1	1.57

Supplementary Table S1: Differentially expressed genes in tumor compared to adjacent non-tumor samples in Lebanese population (Adjusted P<0.05) with a regulation of ≥ +1.5-log^2 fold change.

Gene Name	Symbol	log^2 Fold Change
Delta-Like 1 Homolog (Drosophila)	DLK1	-6.20
WNT Inhibitory Factor 1	WIF1	-4.87
C-Fos Induced Growth Factor	FIGF	-4.60
Oxytocin Receptor	OXTR	-4.02
Tachykinin, Precursor 1	TAC1	-3.88
Chromosome 2 Open Reading Frame 40	C2orf40	-3.74
Collagen, Type VI, Alpha 6	COL6A6	-3.70
Proenkephalin	PENK	-3.69
Myosin Binding Protein C, Slow Type	MYBPC1	-3.55
Lymphatic Vessel Endothelial Hyaluronan Receptor 1	LYVE1	-3.50
Keratin 14, Type I	KRT14	-3.50
Doublecortin	DCX	-3.44
Adiponectin, C1Q And Collagen Domain Containing	ADIPOQ	-3.25
Perilipin 1	PLIN1	-3.12
Protein Tyrosine Phosphatase, Receptor-Type, Z Polypeptide 1	PTPRZ1	-2.96
Leptin	LEP	-2.94
Family With Sequence Similarity 150, Member B	FAM150B	-2.90
Fatty Acid Binding Protein 4, Adipocyte	FABP4	-2.89
Kelch-Like Family Member 13	KLHL13	-2.81
Reelin	RELN	-2.81
Lipoprotein Lipase	LPL	-2.81
Phosphoenolpyruvate Carboxykinase 1 (Soluble)	PCK1	-2.81
Chordin-Like 1	CHRDL1	-2.80
Apolipoprotein D	APOD	-2.74
Transmembrane Protein 132C	TMEM132C	-2.74
LOC100505851	LOC100505851	-2.72
Activin A Receptor, Type IC	ACVR1C	-2.71
Osteoglycin	OGN	-2.71
Immunoglobulin Superfamily, Member 10	IGSF10	-2.67
Lactalbumin, Alpha	LALBA	-2.66
Mab-21-Like 1 (C. Elegans)	MAB21L1	-2.65
Butyrobetaine (Gamma), 2-Oxoglutarate Dioxygenase (Gamma-Butyrobetaine Hydroxylase) 1	BBOX1	-2.59
Serum Deprivation Response	SDPR	-2.57
Parathyroid Hormone-Like Hormone	PTHLH	-2.57
Secretoglobin, Family 3A, Member 1	SCGB3A1	-2.56
R-Spondin 3	RSPO3	-2.55
TIMP Metallopeptidase Inhibitor 4	TIMP4	-2.53

Supplementary Table S2: Differentially expressed genes in tumor compared to adjacent non-tumor samples in Lebanese population (Adjusted P<0.05) with a regulation of ≥ -2.5-log^2 fold change.

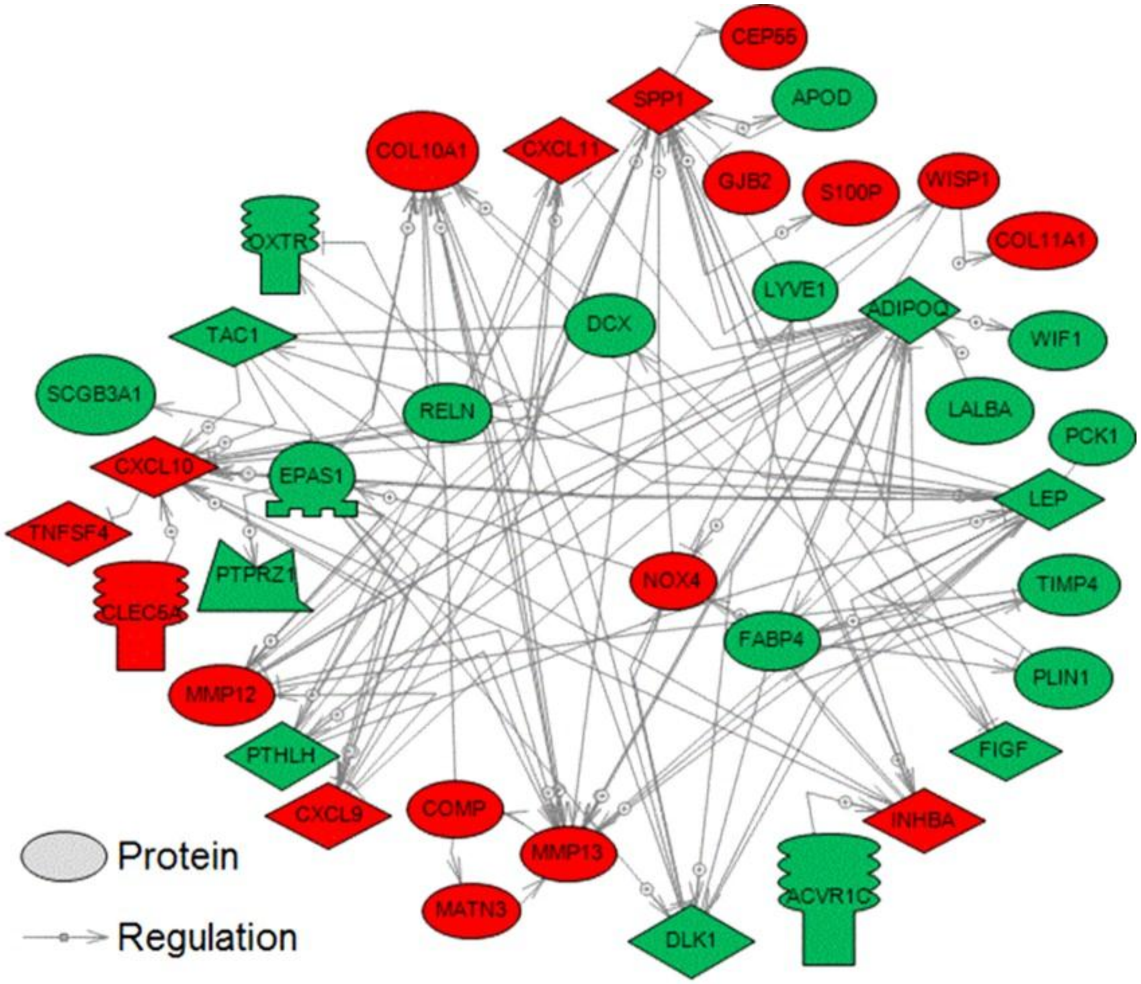

Supplementary Figure S1: Functional relationship network of DEGs between tumor and non-tumor breast tissue, in Lebanese population.

Pathway Studio generated network interactions between most significant DEGs in breast tissue (adjusted P < 0.05, stringency ≥ ± 2-fold change in expression). Upregulated genes are designated in red and downregulated genes in green.

Category	Term	Description	Count	P
Protein Class	PC00074	Chemokine	3	0.0245
	PC00083	Cytochine	6	0.000413
	PC00207	Signaling Molecule	11	0.000867
Molecular Function	GO:0005126	Cytokine Receptor Binding	4	0.00717
	GO:0005125	Cytokine Activity	7	0.000046
	GO:0005102	Receptor Binding	11	0.000276

Supplementary Table S3: The GO functional analysis of the upregulated DEGs, in Lebanese population. Fold Enrichment > 5; P<0.05 by Bonferroni's test.

Gene name	Symbol	Log² Fold Change
PARP1 Binding Protein	PARPBP	3.78
Chloride Intracellular Channel 5	CLIC5	3.71
Werner Helicase Interacting Protein 1	WRNIP1	3.60
Mitochondrial Ribosomal Protein S16	MRPS16	3.16
FXYD Domain Containing Ion Transport Regulator 6	FXYD6	3.06
LIM And Senescent Cell Antigen-Like Domains 1	LIMS1	3.05
Spermatogenesis Associated 25	SPATA25	-3.02
T-Box 19	TBX19	-3.03
Ubiquitin-Conjugating Enzyme E2B	UBE2B	-3.04
EPB41L4A Antisense RNA 2 (Head To Head)	EPB41L4A-AS2	-3.05
USP2 Antisense RNA 1 (Head To Head)	USP2-AS1	-3.05
MET Proto-Oncogene, Receptor Tyrosine Kinase	MET	-3.07
DNA (Cytosine-5-)-Methyltransferase 3 Alpha	DNMT3A	-3.08
Family With Sequence Similarity 3, Member C	FAM3C	-3.08
Ecdysoneless Homolog (Drosophila)	ECD	-3.09
Family With Sequence Similarity 86, Member C1	FAM86C1	-3.11
MFI2 Antisense RNA 1	MFI2-AS1	-3.11
Na+/K+ Transporting ATPase Interacting 3	NKAIN3	-3.17
Chromosome 3 Open Reading Frame 22	C3orf22	-3.17
Platelet Factor 4 Variant 1	PF4V1	-3.20
Immunoglobulin Superfamily, Member 22	IGSF22	-3.20
CAMP-Regulated Phosphoprotein, 19kDa	ARPP19	-3.21
Loricrin	LOR	-3.25
Translocase Of Inner Mitochondrial Membrane 10 Homolog B	TIMM10B	-3.30
Guanylate Cyclase Activator 2B (Uroguanylin)	GUCA2B	-3.33
Adhesion G Protein-Coupled Receptor E5	ADGRE5	-3.36
Transmembrane Protein 97	TMEM97	-3.46
DPP10 Antisense RNA 3	DPP10-AS3	-3.48
Calcium Channel, Voltage-Dependent, L Type, Alpha 1S Subunit	CACNA1E	-3.52
Transmembrane Anterior Posterior Transformation 1	TAPT1	-3.53
LEF1 Antisense RNA 1	LEF1-AS1	-3.54
Olfactory Receptor, Family 1, Subfamily E, Member 1	OR1E1	-3.54
T-Complex 10	TCP10	-3.55
Transcription Elongation Factor B (SIII), Polypeptide 1 (15kDa, Elongin C)	TCEB1	-4.03
Uridine Monophosphate Synthetase	UMPS	-4.06
Benzodiazepine Receptor (Peripheral) Associated Protein 1	BZRAP1	-4.09
Polymerase (RNA) II (DNA Directed) Polypeptide J, 13.3kDa	POLR2J	-4.27
Notch 2 N-Terminal Like	NOTCH2NL	-4.28
Ankyrin Repeat Domain 54	ANKRD54	-4.37
Lactamase, Beta 2	LACTB2	-4.44
Solute Carrier Family 22, Member 14	SLC22A14	-4.57
Ribosomal Protein S12	RPS12	-4.63
Transmembrane Protein 176A	TMEM176A	-5.10
Tumor Necrosis Factor (Ligand) Superfamily, Member 13	TNFSF13	-5.17

Supplementary Table S4: Differentially expressed genes in tumor compared to non-tumor samples in Western populations (Adjusted P<0.05) with a regulation of ≥ ±3-log² fold change.

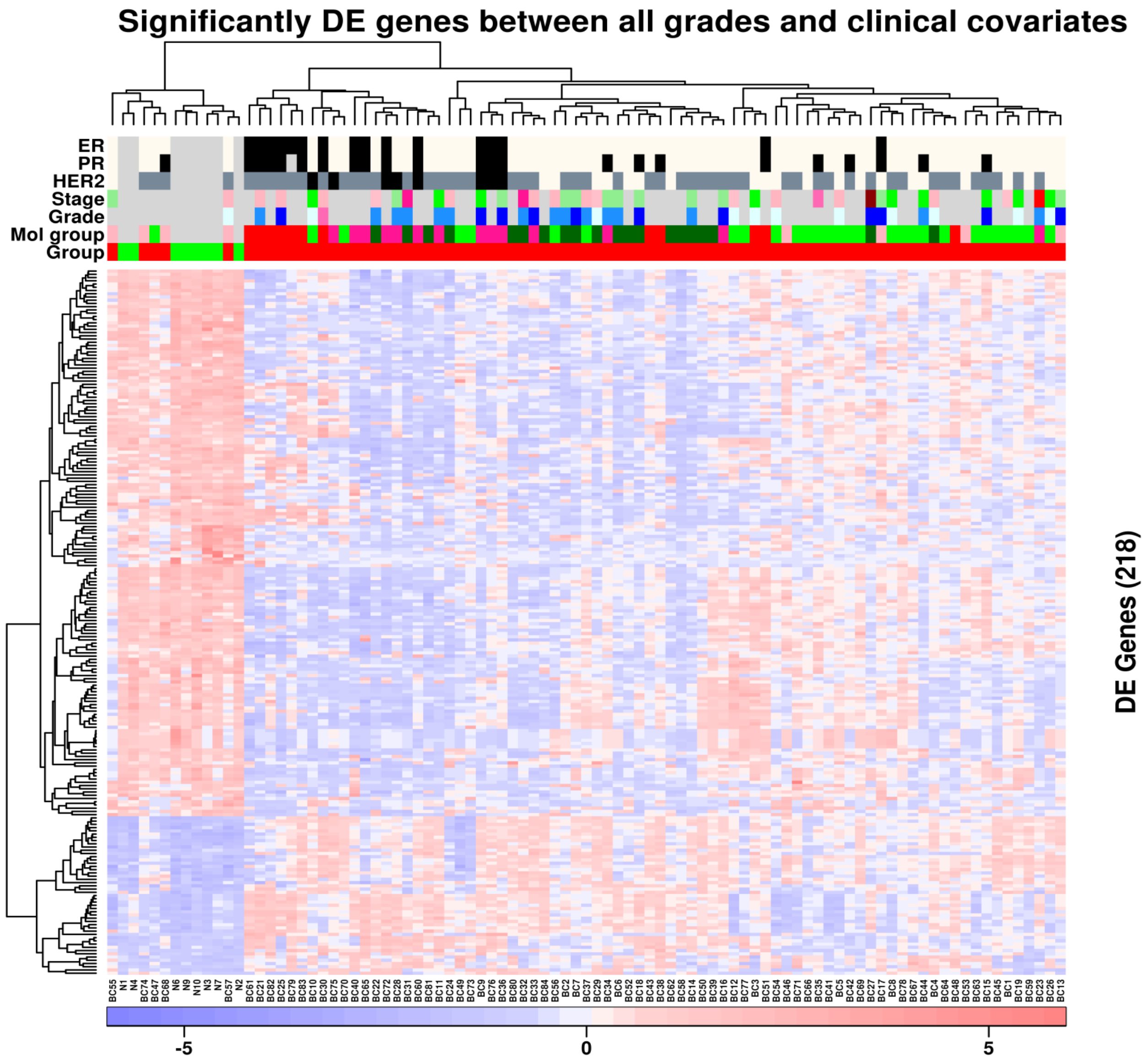

Supplementary Figure S2: Heat map of differentially expressed genes in tumor compared to adjacent non-tumor samples, in Lebanese population.

2-dimentional heat map of significant DEGs between clinical variables (tumor stage and ER/PR/HER2 status) and all molecular subtypes. Heat map of mRNA abundance intensities of the differentially expressed genes in the profiled samples. RMA preprocessed data was transformed to z-scores. The legend represents relative over- (red) and under-expression (blue). The labeling at the bottom represents the normal (N) and tumor samples (BC). Adjusted P<0.05, stringency ≥ ± 3-log^2 fold change in expression. ***Group***: breast cancer = red, normal = green

Grade: NA = light grey, 1 = light cyan, 2 = light blue, 3 = blue, intermediate = pink

Stage: NA = light grey, T1N0 = light green, T1N1 = green, T2N0 = light pink, T2N1 = pink, T2N3 = dark pink, T2N0 = red, T3N1 = dark red, TxN0 = bright pink

ER and PR status: NA = light grey, negative (0) = floral white, positive (1) = black

HER2 status: NA = light grey, negative (0) = floral white, intermediate (1) = dark gray, positive (2) = black

Molecular group: normal = light grey, basal = red, her2 = pink, normal = light pink, luminal A = green, luminal B = dark green

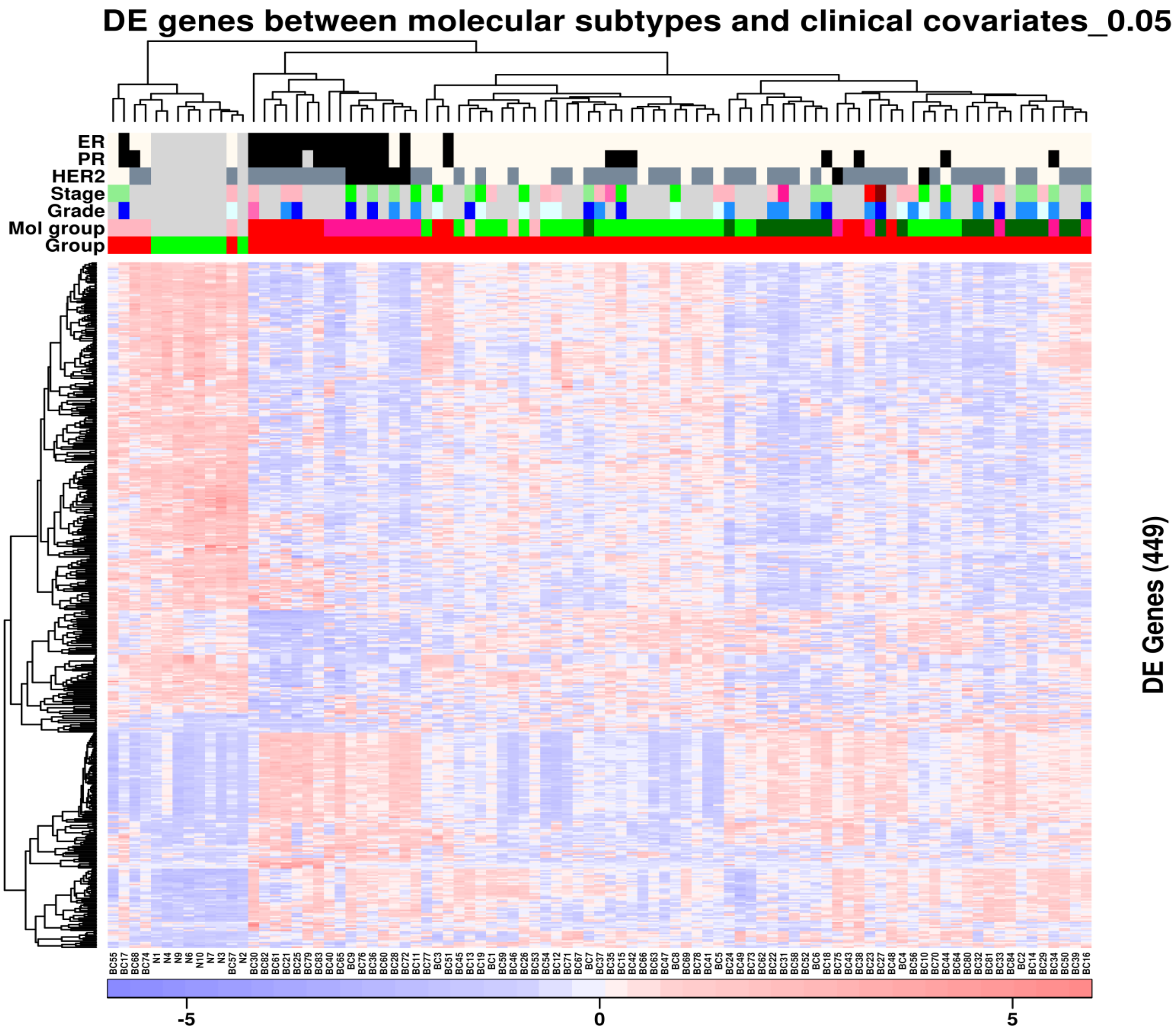

Supplementary Figure S3: Heat map of differentially expressed genes in tumor compared to adjacent non-tumor samples, in Lebanese population.

2-dimentional heat map of significant DEGs between clinical variables (tumor stage and ER/PR/HER2 status) and all molecular subtypes. Heat map of mRNA abundance intensities of the differentially expressed genes in the profiled samples. RMA preprocessed data was transformed to z-scores. The legend represents relative over- (red) and under-expression (blue). The labeling at the bottom represents the normal (N) and tumor samples (BC). Adjusted P<0.05, stringency ≥ ± 3-log^2 fold change in expression. ***Group***: breast cancer = red, normal = green

Grade: NA = light grey, 1 = light cyan, 2 = light blue, 3 = blue, intermediate = pink

Stage: NA = light grey, T1N0 = light green, T1N1 = green, T2N0 = light pink, T2N1 = pink, T2N3 = dark pink, T2N0 = red, T3N1 = dark red, TxN0 = bright pink

ER and PR status*:* NA = light grey, negative (0) = floral white, positive (1) = black

HER2 status: NA = light grey, negative (0) = floral white, intermediate (1) = dark gray, positive (2) = black

Molecular group: normal = light grey, basal = red, her2 = pink, normal = light pink, luminal A = green, luminal B = dark green

Clinical characteristics	Number (%)
Age (years)	
**<40**	15 (18)
**40-49**	18 (21)
**50-69**	42 (50)
**70+**	7 (8)
**Undetermined**	2 (3)
Menopause status	
**Premenopausal**	37 (44)
**Postmenopausal**	46 (55)
**Undetermined**	1 (1)
Tumor grade	
**I**	20 (24)
**II**	30 (36)
**III**	24 (28)
**Undetermined**	10 (12)
Molecular subtype	
**Normal-like**	8 (10)
**Luminal A**	29 (34)
**Luminal B**	20 (24)
**Basal**	12 (14)
**HER2**	14 (18)
Estrogen Receptor	
**Negative**	16 (20)
**Positive**	67 (78)
**Undetermined**	1 (2)
Progesterone Receptor	
**Negative**	23 (27)
**Positive**	59 (70)
**Undetermined**	2 (3)
HER2	
**Negative**	52 (62)
**Positive**	8 (10)
**Undetermined**	24 (28)
Family history of breast cancer	
**Yes**	37 (44)
**No**	46 (55)
**Undetermined**	1 (1)
Family history of ovarian cancer	
**Yes**	3 (4)
**No**	79 (94)
**Undetermined**	2 (2)

Supplementary Table S5: Characteristics of Lebanese breast cancer patients.

Gene Symbol	Full name		Oligonucleotide sequences
FIGF	c-Fos Induced Growth Factor	F	CTTCTGGAGAATGCCTTTTG
		R	AATGTGGACTGAGATGATCG
ADIPOQ	Adiponectin C1Q And Collagen Domain Containing	F	CTTTGCCTACCACATCACAG
		R	CTCTCCTTCCCCATACACC
LEP	Leptin	F	ACACCAAAACCCTCATCAAG
		R	CGTTTCTGGAAGGCATACTG
PTHLH	Parathyroid Hormone-Like Hormone	F	AGTCCATCCAAGATTTACGG
		R	GTATCTGCCCTCATCATCAG
MMP13	Matrix Metallopeotidase 13	F	CAGTGGTGGTGATGAAGATG
		R	CCTCTAAGCCGAAGAAAGAC
S100P	S100 Calcium-Binding Protein P	F	GCACCATGACGGAACTAGAG
		R	ATTTATCCACGGCATCCTTG
COL10A1	Collagen Type X Alpha 1	F	TATGCTGCCACAAATACCC
		R	CCTTGCTCTCCTCTTACTGC
FOSL1	FOS-like Antigen 1	F	CGCCCTGTACCTTGTATCTC
		R	GCTGCTGCTGCTACTCTTG
PGK1	Phosphoglycerate kinase 1	F	CAAGAAGTATGCTGAGGCTGTCA
		R	CAAATACCCCCACAGGACCAT

Supplementary Table S6: Oligonucleotide sequences used in confirmatory quantitative real-time PCR analysis.
